# The relationship between nurses’ stressors and work engagement among Chinese nurses: the chain-mediating effect of career resilience and team job crafting

**DOI:** 10.3389/fpsyg.2026.1689528

**Published:** 2026-08-31

**Authors:** Xiangjie Sun, Qingsheng Zhao, Yu Li, Hong Wang, Yongli Li, Yanfei Hu, Changmin Zhang

**Affiliations:** Emergency Department Outpatient Observation Room, Qilu Hospital of Shandong University, Jinan, China

**Keywords:** career resilience, mediation effect, nurse stressors, team job crafting, work engagement

## Abstract

**Background:**

Amid the ongoing shortage of nursing personnel, increasing attention has been paid to nurses’ work engagement, as their role is a high-stress profession. Work engagement among nurses not only reflects their efficiency but is also closely linked to the quality of patient care. This study analyzes the unexplored relationship between nurses’ stressors and work engagement, investigates the multiple mediating roles of career resilience and team job crafting and offers important insights for boosting work commitment.

**Methods:**

This cross-sectional study was conducted using convenience sampling at three tertiary hospitals in Shandong Province, China, from July to August 2024. A total of 890 questionnaires were distributed, and 820 valid questionnaires were returned. Data were collected using a demographic characteristics questionnaire, career resilience scale, team job crafting scale, and work engagement scale. Descriptive statistics, Pearson correlation analysis, and chain mediation analysis were conducted using SPSS 26.0 and AMOS 26.0.

**Results:**

There were significant correlations between nurses’ stressors, career resilience, team job crafting, and work engagement (*r* = −0.28 ∼ 0.40, *P* < 0.01). Nurses’ stressors exhibited a significant direct effect on work engagement (β = −0.31, Confidence Interval: −0.404 ∼−0.227). The mediation analysis revealed that nurses’ stressors also influenced work engagement through three statistically significant indirect pathways, which collectively represented 46% of the total effect. They included (a) the mediating effect of career resilience (β = −0.141), (b) the mediating effect of team job crafting (β = −0.062), and (c) the chain mediating effect between career resilience and team job crafting (β = −0.025).

**Conclusion:**

Nurses’ stressors had a direct negative effect on work engagement among nurses. Meanwhile, career resilience and team job crafting played a chain-mediating role between nurses’ stressors and work engagement. Consequently, hospital administrators should focus on stress management, career resilience, and team job crafting to enhance engagement.

## Background

Nurses play a vital role in healthcare provision, particularly as demand for services grows in tandem with rapid socioeconomic development and population aging. They constitute the largest segment of the healthcare workforce, representing 57% of health professionals worldwide (Marć et al., 2019; World Health Organization (WHO), 2025). However, there is currently an acute global shortage of approximately 6 million nurses (Alshehry et al., 2019), and the nursing workforce is unable to expand rapidly enough to satisfy healthcare demands. In addition to accelerating training of new nurses and reducing turnover among current staff, hospital administrators must prioritize enhancing existing nurses’ work engagement. This approach is vital for alleviating the shortage of nursing professionals and improving patient care quality (Cai et al., 2023).

Work engagement is a fulfilling and positive attitude characterized by dedication, absorption, and vigor (Schaufeli et al., 2002). High levels of nurses’ work engagement are linked to several positive outcomes, including better patient experiences, higher job performance, better-rated nursing care quality, higher patient satisfaction, and lower absenteeism (Alluhaybi et al., 2023; Aungsuroch et al., 2024; Dempsey and Assi, 2018). Furthermore, it is necessary to improve work engagement to ensure nursing care quality, especially given that a previous study found a significant decline in nurses’ work engagement compared to pre-epidemic levels (Penturij-Kloks et al., 2023). Beyond individual-level factors, work engagement is also shaped by team- and organization-level contextual factors. For instance, organizational cultural tightness – the strength of social norms and the degree of sanctioning within an organization – has been shown to influence work engagement, with transformational leadership moderating this relationship (Song et al., 2022). Similarly, team-level cultural tightness can affect employee outcomes through cross-level mechanisms (Shi et al., 2023). These findings highlight the importance of considering contextual factors alongside individual characteristics when examining work engagement. Therefore, motivating nurses is critical.

Various studies have demonstrated that work stressors affect nurses’ work engagement. Zhang’s research indicated that work stressors or work overload were significantly and negatively related to work engagement (Zhang et al., 2022). However, most current studies focus only on the single nurse work stressor’s (e.g., individual, organizational, or work environment, etc.) effect on work engagement (Mantzorou et al., 2020; Zhang et al., 2022). Nursing is known to be a high-stress profession, where nurses need to cope with high work demands and often experience multiple stressors at the same time (e.g., work-related and time allocation problems) (Jun et al., 2021; Chen et al., 2020; Jin et al., 2023). Therefore, it is necessary to explore the impact of comprehensive stressors on nurses’ work engagement. “Nurses’ stressors” refers to the factors that nurses perceive as relevant to the generation of job stress due to work: the nursing profession and work-related problems, time allocation and workload problems, the work environment and equipment problems, patient care problems, and management and interpersonal relationship problems (Hua, 2007). Exposure to multiple occupational stressors may contribute to job stress and job burnout, which may subsequently affect patient outcomes and decrease nurses’ job satisfaction (Bautista et al., 2020; Sun et al., 2017). In addition, according to stress cybernetic theory, when an individual suffers multiple work stressors simultaneously, their ability to work decreases, which ultimately leads to work engagement decline (Dewe et al., 2012). Therefore, we hypothesized that nurses’ stressors would negatively predict work engagement.

The Job Demands-Resources (JD-R) model suggests that individuals’ personal resources can reduce the negative impact of comprehensive work stressors on job performance and promote positive organizational outcomes, such as increased individual work engagement (Bakker and Demerouti, 2017). Current studies focus on personal resources like psychological capital and psychological resilience, but nurses’ stressors arising from specific occupational settings, along with psychological capital or resilience, do not reflect the interactions between individuals’ psychological attributes and occupational contexts (Aqtam et al., 2023; Aqtam et al., 2025; Liu et al., 2021; Mealer et al., 2017). Career resilience in nurses refers to the psychological trait of coping positively with occupational dilemmas in the face of unpromising occupational contexts (Jiang et al., 2024). Previous research has observed that work stressors are associated with higher levels of psychological resilience, which, in turn, are associated with work engagement among non-healthcare occupational populations (Black et al., 2017). Thus, we hypothesized that career resilience mediates the relationship between stressors and work engagement among nurses.

When individuals encounter various work stressors that lead to unfavorable working conditions, how can they motivate themselves to work? Wrzensniewski and Dutton proposed the concept of job crafting, which emphasizes an individual’s subjective initiative at work. Job crafting refers to the physical and cognitive changes that individuals experience during work tasks that encourage positive work behaviors and goal accomplishment (Jane, 2002). In many occupational contexts, job crafting occurs at both the individual and team levels because employees in work teams often experience common events, participate in the same work processes, and influence each other (Tims et al., 2013). However, current research has focused mainly on job crafting at the individual level among nurses, ignoring team job crafting (Han, 2023; Khattab et al., 2023). Team job crafting is a new concept defined as the extent to which team members combine efforts to increase structural and social job resources and decrease hindering job demands (Tims et al., 2013). In this study, team job crafting was assessed via individual nurses’ perceptions of their team’s collective behaviors – that is, each nurse reported their own perception of the extent to which their team engaged in job crafting. While team job crafting is conceptually a team-level construct, individual-level perceptual measurement is appropriate when the research focus is on how individual employees interpret and respond to team-level phenomena in shaping their personal work outcomes (Tims et al., 2013). With the advancement of modern medicine, complexity and instability have characterized the nursing work conditions of hospitals, and clinical nurse care is now usually delivered through team nursing (Goh et al., 2020). In team-based learning, continuous patient care is facilitated by mutually supportive workload management, more effective communication, enhanced interpersonal relationships, and strong team orientation, all of which are key for achieving team goals and delivering high-quality care (Barton et al., 2018; Iida et al., 2021). Additionally, the JD-R model incorporates aspects of job crafting, whereby individuals, when faced with various work stressors – such as excessive job demands or mismatched resources – engage in adjusting physical and cognitive changes, thereby promoting positive outcomes (e.g., work engagement) (Tims and Bakker, 2010). Subsequently, team job crafting is expected to be more effective in reducing work stress by increasing resources, crafting work boundaries, and ultimately promoting work engagement (Qi et al., 2016). Studies in different non-healthcare occupational populations have found a positive correlation between team job crafting and work engagement (Llorente-Alonso and Topa, 2019; McClelland et al., 2014). In the nursing context, Iida et al. recently confirmed a direct positive association between team job crafting and work engagement using a prospective cohort design (Iida et al., 2024). However, whether team job crafting serves as a mediator between nurses’ stressors and work engagement remains unknown. Accordingly, we hypothesized that team job crafting would mediate the relationship between nurses’ stressors and work engagement.

As discussed, career resilience and team job crafting may independently act as mediators between nurses’ stressors and work engagement, but their interrelationship as moderators needs further clarification. Previous studies have shown that personal resources significantly predict job resources, suggesting that the relationship between nurses’ stressors and work engagement may first be influenced by career resilience and later by team job crafting. Therefore, we hypothesized that career resilience and team job crafting play a chain mediation role between nurses’ stressors and occupational dedication.

**FIGURE 1:**
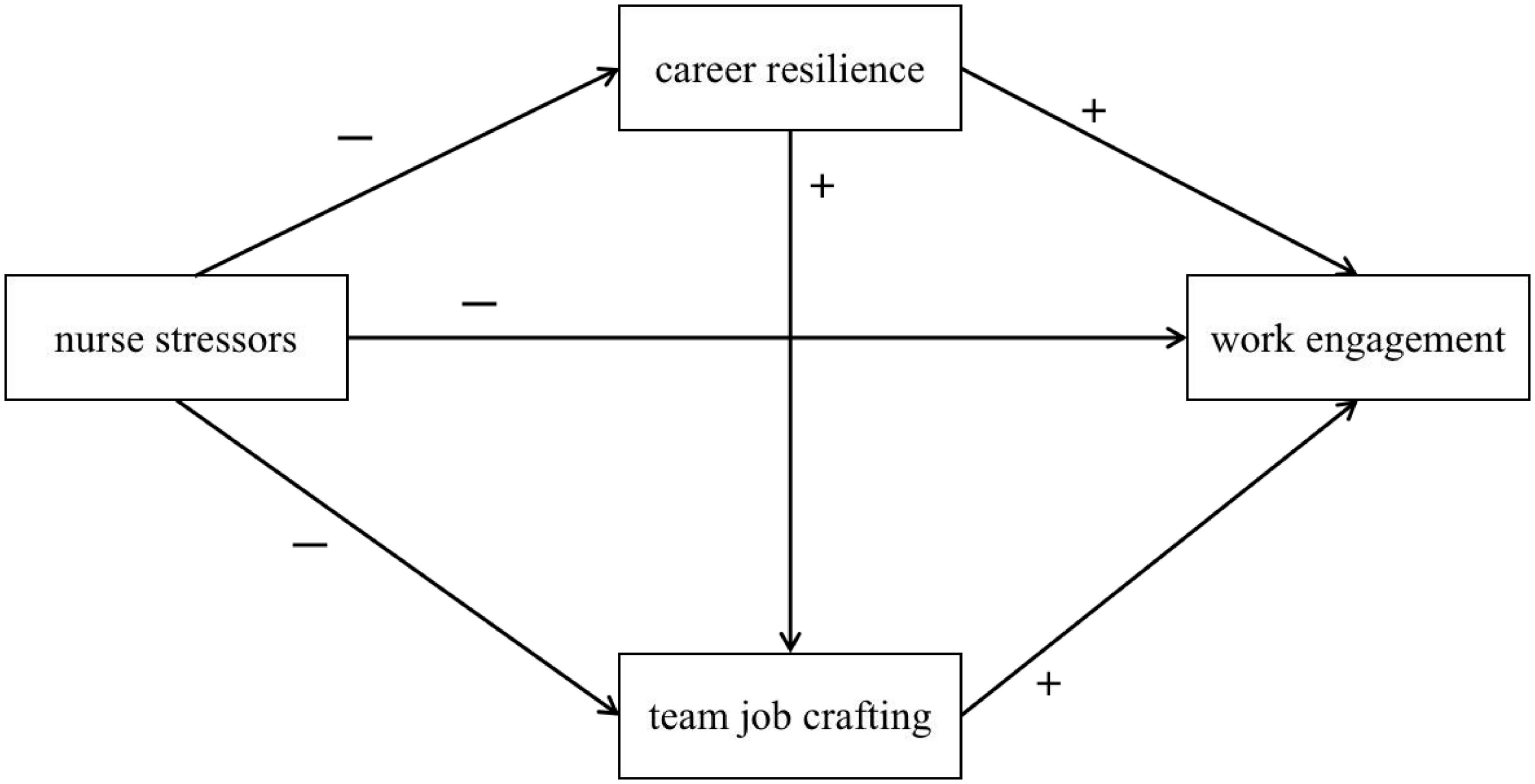
Research framework.

Based on the current literature and the JD-R model, few studies have explored the relationships among nurses’ stressors, career resilience, team job crafting, and work engagement. Understanding the potential mechanisms by which nurses’ stressors influence work engagement is critical for informing future interventions. Therefore, to fill existing knowledge gaps, this study investigated the multiple mediating effects of career resilience and team job crafting on the relationship between nurses’ stressors and work engagement, thereby offering a theoretical basis for improving nurses’ professional engagement. The hypothesized conceptual model for this study is shown in Figure 1.

## Methods

### Study design

This cross-sectional survey was conducted from July to August 2024 to explore the relationships between nurses’ stressors, career resilience, team job crafting, and work engagement among nurses.

### Participants

We recruited nurses from three tertiary hospitals in Shandong Province, China, using convenience sampling. The nurses’ inclusion criteria were as follows: (1) valid nursing practice certificates, (2) a valid period of registration, (3) over 1 year of clinical work experience, and (4) informed consent and voluntary survey participation. The exclusion criteria included nurses absent during the survey period and nursing interns, outsiders, or those on training leave.

### Sample size

Structural equation modeling (SEM) and maximum likelihood estimation were used to examine the relationships among variables. The sample size was established using an N: q ratio of 10:1, where N is the minimum number of cases required and q is the number of items to be estimated (Jackson, 2003). In this study, q was 71, and the minimum sample size was 710. Considering 20% invalid questionnaires, a sample size of 852 was required.

### Data collection

A paper-version questionnaire was used and collected face-to-face with the help of hospital administrators. In the pre-survey phase, the participants were briefed about the purpose and significance of the study and assured that the collected data would only be used for research and would be anonymized throughout. The participants were allowed to participate and withdraw at any time during the survey. A total of 890 questionnaires were distributed, and 875 were returned, with a 98.3% response rate. A total of 820 valid questionnaires were returned after excluding 55 invalid ones, with an effective recovery rate of 92.1%.

### Measures

#### Demographic characteristics

The self-designed demographic characteristic questionnaire included age, sex, education level, marital status, and professional title.

#### Nurses’ stressor

Nurses’ stressors were assessed using the Chinese version of the Nurse Stressor Scale.

This scale was redeveloped by Li based on the actual situation in China, with reference to the Nursing Work Stress Scale compiled by Grey in the United States and the Nurse Work Stressor Scale compiled by Wheeler in the United Kingdom (Gray-Toft and Anderson, 1981; Hua, 2007; Wheeler and Riding, 1994). The Chinese version of the Nurse Stressor Scale has 35 items and contains five dimensions: nursing profession and work, time allocation and workload, work environment and equipment, patient care, management, and interpersonal relationships. A 4-point Likert scale was used, with scores of 1–4 representing “no stress” to “severe stress”; higher scores indicate a higher degree of stress caused by nurses’ stressors. In this study, the Cronbach’s α for the scale was 0.88.

#### Career resilience

We selected three dimensions to assess career resilience among nurses based on their actual working situation (Zhang, 2016), utilizing the Career Resilience Scale for Chinese College Counselors developed by Zhang. Although this scale was originally developed for college counselors, its core dimensions – emotional stability, self-adjustment ability, and social support resources – represent generic psychological attributes that are conceptually relevant across occupational contexts. Previous studies have successfully applied this scale to other professional populations, including healthcare workers. The Career Resilience Scale consists of seven items and three dimensions: emotional stability, self-adjustment ability, and social support resources. This is a 5-point Likert scale, with 1 being “strongly disagree” and 5 being “strongly agree.” Higher scores indicated higher levels of career resilience. To verify the construct validity of this scale in our nursing sample, we conducted a confirmatory factor analysis. The results demonstrated excellent model fit: CFI = 0.993, TLI = 0.990, GFI = 0.996, and RMSEA = 0.010, supporting the three-dimensional structure of the scale in this sample. The Cronbach’s α of this scale was 0.77.

#### Team job crafting

The Team Job Crafting Scale developed by Maco and translated by Zhang was used to assess nurses’ team job crafting among nurses (Fang, 2023; Iida et al., 2021). This scale consists of two dimensions: task crafting (four items) and cognitive and relational crafting (nine items). This scale uses a 5-point Likert scale format, where 1 indicates “never” and 5 indicates “always.” In this study, team job crafting was measured at the individual perception level – that is, rather than aggregating individual responses to the team level, each nurse reported their own perception of the extent to which their team collectively engaged in job crafting behaviors. While team job crafting is conceptually a team-level construct, individual-level perceptual measurement is appropriate for our research question, as we examine how individual perceptions of team-level phenomena mediate the relationship between personal stressors and individual work engagement. This approach is consistent with established practices in team-level research, wherein individual members’ perceptions are commonly used as indicators of team constructs when the analytical focus is on individual-level outcomes. The Cronbach’s α of this scale was 0.96.

#### Work engagement

The Work Engagement Scale developed by Schaufeli and translated by Zhao was used to assess the nurses’ work engagement (Schaufeli et al., 2002; Zhao, 2011). This scale includes nine items across three dimensions: vigor, absorption, and dedication. This 7-point Likert scale ranges from 0 being “never” and 6 being “always.” Higher scores indicated higher levels of work engagement. The Cronbach’s α of this scale was 0.93.

### Statistical analysis

SPSS 26.0 and AMOS 26.0 were used for the statistical analysis. Descriptive statistics (means, percentages, frequencies, etc.) were used to measure participants’ socio-demographic characteristics, nurses’ stressors, career resilience, team job crafting, and work engagement. Harman’s single-factor test was conducted using unrotated exploratory factor analysis to assess common method bias (Podsakoff et al., 2003). To further test for common method bias, we conducted a Common Latent Factor (CLF) analysis using AMOS 26.0. One-Way Analysis of Variance (ANOVA) was used to examine differences in work engagement scores across various demographic variables. Pearson correlation analysis was used to examine the bivariate relationships between the four variables. In addition, SEM was used to develop a path model with confirmatory modeling based on robust maximum likelihood. Nurses’ stressors were the independent variables, work engagement was the dependent variable, and career resilience and team job crafting were the mediating variables in the model. The direct and indirect effects were calculated by bias-corrected bootstrap of 5000 replications with a 95% confidence interval (CI). *P* < 0.05 was statistically significant. The model fit included the following indices: root mean square error of approximation (RMSEA < 0.08), goodness-of-fit index (GFI > 0.9), comparative fit index (CFI > 0.9), and Tucker–Lewis index (TLI > 0.9) (Browne and Cudeck, 1992).

### Ethics considerations

This study was approved by the Ethics Committee of Qilu Hospital of Shandong University (Ethics No. KYLL-202405-001-1). All participants provided informed consent before participating in the survey, remained anonymous, and participated voluntarily in the study.

## Results

### Common method bias

To rule out the potential influence of common method bias associated with self-report questionnaires, this study conducted Harman’s one-factor test. All items from the four scales were included in an unrotated exploratory factor analysis, which identified nine factors with eigenvalues greater than 1. The first principal factor explained 33.90% of the variance, which is below the critical threshold of 40%.

To further verify this result with a more rigorous approach, we performed a Common Latent Factor (CLF) analysis using AMOS 26.0. Specifically, we added a common latent factor to the measurement model, allowing all observed indicators to load onto this factor, with equal loadings constrained, its variance fixed to 1, and its covariances with the substantive latent factors set to zero.

The CLF-controlled model demonstrated adequate fit: CFI = 0.925, GFI = 0.915, TLI = 0.906, and RMSEA = 0.075. Compared with the final modified model without the CLF (CFI = 0.920, GFI = 0.921, TLI = 0.913, RMSEA = 0.076), the changes in fit indices were minimal (ΔCFI = 0.005, ΔTLI = −0.007, ΔRMSEA = −0.001), all well below the recommended thresholds (ΔCFI ≤ 0.01, ΔRMSEA ≤ 0.05). Moreover, the standardized factor loadings of the observed items on their corresponding substantive factors showed no substantial decreases, with the maximum difference being less than 0.200. These results indicate that common method bias does not pose a serious threat to the validity of findings.

### Participants’ characteristics

A total of 820 clinical nurses participated in the survey: 663 were women, and 759 had a bachelor’s degree. The mean age of the sample was 31.69 ± 6.57 (range = 20–50) years. Of the participants, 62.7% of the were married, 43.4% worked in the emergency department, and 62.4% had worked for under 10 years. See Table 1 for details.

**TABLE 1:** Work engagement scores of nurses according to sociodemographic characteristics of the sample (*N* = 820)

Variables	N (%)	M (SD)	t/F	*P*
Gender				
Women	663 (80.85)	37.17 (11.55)	2.771	0.096
Men	157 (19.15)	35.25 (13.17)		
Age (years)				
≤25	186 (22.68)	34.47 (13.78)	4.033	0.003
26–30	183 (22.32)	36.07 (12.76)		
31–35	379 (46.22)	39.58 (11.36)		
36–40	64 (7.80)	45.88 (4.49)		
41–50	8 (0.98)	35.62 (12.89)		
Educational level				
Associated degree	21 (2.56)	40.35 (13.38)	1.405	0.246
Bachelor’s degree	759 (92.56)	35.48 (12.99)		
Master’s degree or above	40 (4.88)	35.92 (10.44)		
Marital status				
Married	514 (62.68)	33.54 (13.01)	12.465	<0.001
Single	306 (37.32)	36.84 (12.68)		
Years of clinical working				
1–10	513 (62.56)	35.39 (12.84)	3.403	0.017
11–20	252 (30.73)	35.07 (13.19)		
21–30	46 (5.61)	39 (11.65)		
31–40	9 (1.10)	46.44 (5.50)		
Department				
Internal medicine	178 (21.71)	33.25 (13.38)	4.094	0.003
Surgery	56 (6.83)	38.95 (11.51)		
Emergency department	356 (43.41)	36.57 (12.28)		
Intensive care unit	177 (21.59)	36.13 (12.68)		
Others	53 (6.56)	31.96 (15.55)		
Professional title				
Primary	546 (66.59)	35.13 (12.90)	2.348	0.126
Intermediate or senior	274 (33.41)	36.6 (12.83)		

M means, SD standard deviation.

### Nurse stressor, career resilience, team job crafting, and work engagement correlation

The scores of nurse stressor, career resilience, team job crafting, and work engagement were 87.83 ± 8.57, 22.89 ± 2.89, 30.98 ± 8.97, and 35.59 ± 12.84, respectively. Pearson correlation analysis was conducted to identify potential correlations between the four variables. First, nurses’ stressors were negatively correlated with career resilience (r = −0.283, *P* < 0.001), team job crafting (r = −0.349, *P* < 0.001), and work engagement (r = −0.475, *P* < 0.001). Additionally, career resilience was positively correlated with team job crafting (r = 0.279, *P* < 0.001) and work engagement (r = 0.393, *P* < 0.001). Third, team job crafting was positively correlated with work engagement (r = 0.403, *P* < 0.001). The results of the descriptive analysis of the variables and the Pearson correlation coefficients are presented in Table 2.

**TABLE 2:** Mean, standard deviation (SD), and correlations of the study variables.

Variables	M (SD)	1	2	3	4
1 Nurses’ stressors	87.83 (8.57)	1	1	1	1
2 Career resilience	22.89 (2.89)	−0.283***			
3 Team job crafting	30.98 (8.97)	−0.349***	0.279***		
4 Work engagement	35.59 (12.84)	−0.475***	0.393***	0.403***	

***P < 0.001.

### Mediation effect analysis of the hypothetical model

**FIGURE 2:**
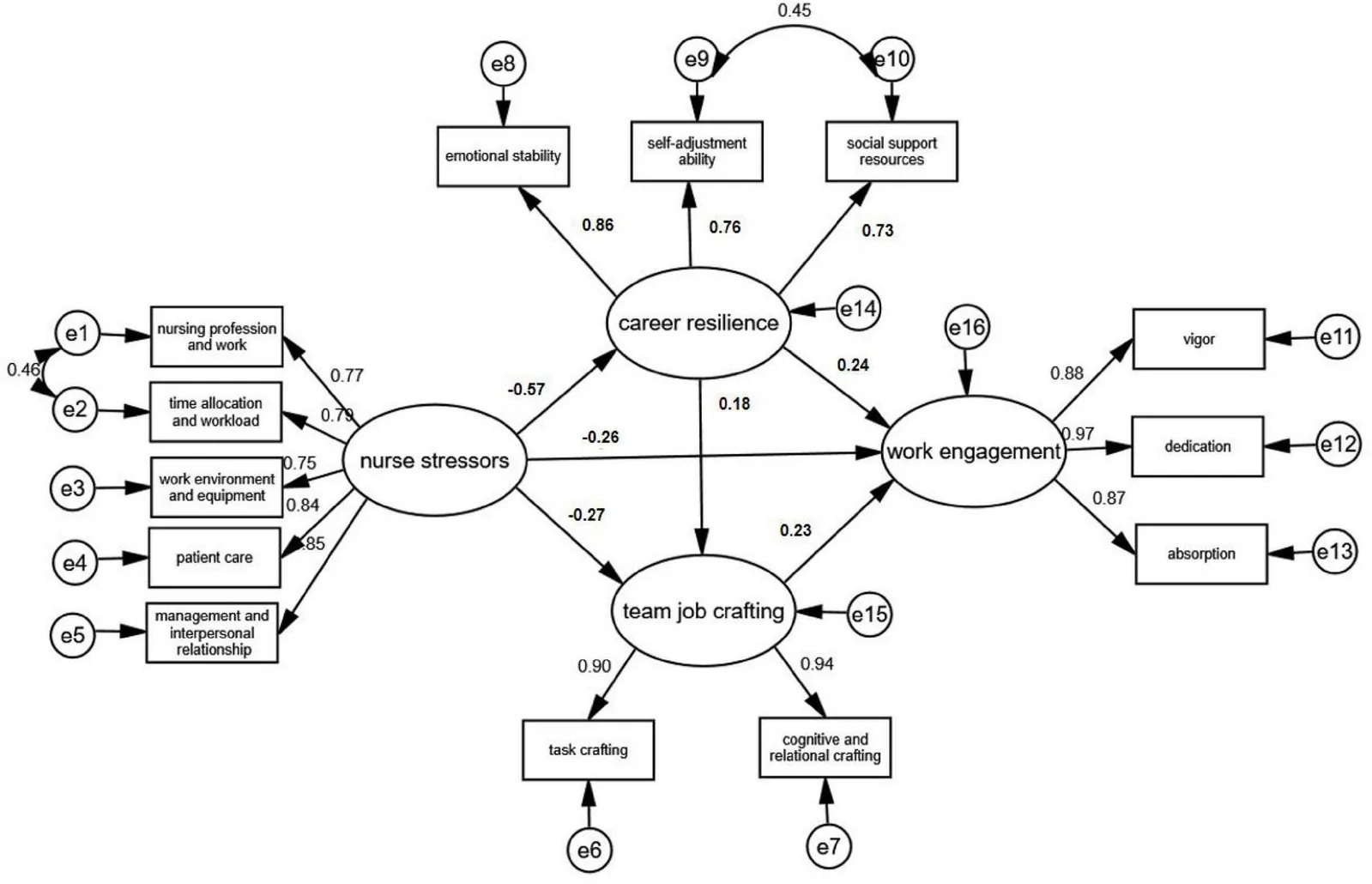
Path diagram for the relationship among nurses’ stressors, career resilience, team job crafting, and work engagement.

To exclude the potential influence of sociodemographic variables on work engagement, we first conducted one-way ANOVA with work engagement as the dependent variable, examining gender, age, education level, marital status, years of clinical working, department, and professional title. The results showed that age, marital status, years of clinical working, and department each had a statistically significant effect on work engagement (*P* < 0.05) (see Table 1). Therefore, these four sociodemographic variables were included as controls in the mediation analysis. The standardized estimates of the path coefficients for each variable are shown in Figure 2. The results of the SEM analysis of the hypothetical model indicated that the data failed to support the theoretical model (see Table 3). Furthermore, the modification index indicated that two pairs of covariance parameters should be included to account for the relationships between nursing profession and work and time allocation and workload, self-adjustment stability and social support resource. Given that these relationships align with theoretical considerations, covariance parameters were included in the model. In the final modified model, the data fit the model (CFI = 0.92, GFI = 0.921, TLI = 0.913, and RMSEA = 0.076).

**TABLE 3:** Goodness-of-fit statistics for the initial model and the modified model.

Steps	Model description	*P*	X^2^/df	GFI	CFI	TLI	RMSEA
1	Initial model	0.000	7.331	0.892	0.904	0.905	0.088
2	Add covariance between e1 and e2	0.000	6.611	0.906	0.916	0.916	0.083
3	Add covariance between e9 and e10	0.000	6.023	0.914	0.925	0.925	0.078

e1: the error terms of nursing profession and work (a dimension of nurses’ stressors); e2: the error terms of time allocation and workload (a dimension of nurses’ stressors); e9: the error terms of self-adjustment stability (a dimension of career resilience); e10: the error terms of social support resource (a dimension of career resilience). *RMSEA* root mean square error of approximation, *TLI* Tucker-Lewis index, *CFI* comparative fit index; *GFI* good-of-fit index.

Table 4 presents the direct and indirect effects of nurses’ stressors on work engagement with career resilience and team job crafting as mediators. SEM revealed significant regression and correlation paths. Furthermore, the results showed that career resilience and team job crafting served as significant mediators, accounting for 46% of the total effect (−0.492), along with a total indirect effect of −0.227. The mediating effect in this study consisted of three paths. Firstly, the coefficient of the indirect effect of nurses’ stressors through career resilience on work engagement was −0.141 (*P* < 0.01); subsequently, the coefficient of the indirect effect of nurses’ stressors through team job crafting on work engagement was −0.062 (*P* < 0.01). Third, the indirect pathways of nurses’ stressors through career resilience and team job crafting on work engagement were −0.025 (*P* < 0.01).

**TABLE 4:** The direct and indirect effects of nurses’ stressors on work engagement.

Effects		Point estimate	95% bias-corrected CI
Indirect effect	Nurses’ stressors → career resilience → work engagement (Ind1)	−0.141	(−0.206, −0.084)**
	Nurses’ stressors → team job crafting → work engagement (Ind2)	−0.062	(−0.098, −0.036)**
	Nurses’ stressors → career resilience → team job crafting → work engagement (Ind3)	−0.025	(−0.045, −0.010)**
Direct effect	Nurses’ stressors → work engagement	−0.264	(−0.375, −0.0148)**
Total indirect effect	Ind1 + Ind2 + Ind3	−0.228	(−0.302, −0.167)**
Total effect	Indirect effect + direct effect	−0.492	(−0.593, −0.421)**

**P < 0.01.

## Discussion

This study aimed to investigate work engagement among nurses and identify how nursing stressors were associated with it. Extending prior research that focused on the direct association between team job crafting and work engagement (Iida et al., 2024), to our knowledge, this study is the first to examine the chain mediating roles of career resilience and team job crafting in the relationship between nurses’ stressors and work engagement among nurses. The results were consistent with a direct association between stressors and work engagement among nurses. Meanwhile, this study found that nurse stressors were associated with work engagement among nurses through three pathways: (a) nurses’ stressors → career resilience → work engagement, (b) nurses’ stressors → team job crafting → work engagement, and (c) nurses’ stressors → career resilience → team job crafting → work engagement. These three pathways accounted for 29%, 12%, and 5% of the total effect, respectively.

For Hypothesis 1, this study found that nurses’ stressors were directly associated with work engagement, showing a negative correlation with work engagement among nurses. This result is consistent with that reported by Cabrera-Aguilar, where higher levels of stressors were associated with lower levels of work engagement (Cabrera-Aguilar et al., 2023). As the largest professional group in the healthcare sector, nurses frequently encounter multiple stressors at work, including patient care problems, work environments and equipment, time constraints, and the tedious task of completing nursing documentation. According to the stress cybernetic theory, when nurses frequently experience excessive comprehensive stressors, this is theorized to be associated with a decline in work ability, which may reduce the efficiency of coping with patients and may be associated with a decrease in the level of work engagement (Sun et al., 2017). Considering the prevalence of nurses’ work stress, this result highlights the necessity of paying attention to nursing work stressors to potentially improve work engagement among nurses.

For Hypothesis 2, this study found that career resilience served as a mediator in the relationship between nurses’ stressors and work engagement. In short, the results were consistent with the pattern that when nurses experience high stress levels, those with high career resilience tend to be better able to cope with the negative effects of multiple stressors, and this coping was associated with higher work engagement. This finding is consistent with the JD-R model (Bakker and Demerouti, 2017). Career resilience in nurses serves as a positive psychological trait within personal resources, representing both individual psychological traits and the interaction between these characteristics and the occupational environment. Nurses with high career resilience possess rich psychological resources, better social skills, and stronger environmental adaptability. When faced with multiple dynamic nursing stressors, they are more likely to maintain emotional stability, expand their self-adjustment ability, and enhance social support resources, which in turn are associated with improved work efficiency (Liu et al., 2021). Specifically, the emotional stability and self-adjustment abilities of nurses with higher career resilience were associated with a greater ability to promptly adjust their cognition, emotions, and coping strategies when handling stressors. They may effectively regulate the relationship between resource depletion and work-related conflict, helping nurses establish strong psychological barriers and adeptly resist negative external influences (Han et al., 2024). This finding also connects career resilience with the broader literature on career-related individual differences. Although career resilience is distinct from constructs such as career adaptability and proactive personality, these characteristics similarly influence how individuals respond to occupational challenges. Previous studies have shown that career-related resources may translate into work engagement, job satisfaction, and performance through adaptive responses and improved person–job fit, and that these effects may depend on individual proactivity (Kaur and Kaur, 2020; Kaur and Kaur, 2021; Rudolph et al., 2017). The present findings extend this literature by suggesting that career resilience may be translated into work engagement partly through nurses’ collective job-crafting behaviors. Additionally, social support resources associated with higher career resilience may help nurses proactively address work-related stressors. Individuals may actively seek assistance from colleagues, organizations, and friends within their social networks and consciously attempt to implement various solutions to resolve work-related challenges, enabling them to confidently navigate occupational difficulties. Therefore, when experiencing stressors, nurses with high career resilience tend to be associated with exhibiting stronger levels of work engagement. From a practical perspective, the career resilience pathway contributed the largest share (29% of the total effect). This finding suggests that career resilience may be a promising intervention target, although the proportion of the indirect effect alone does not establish that it is the most efficient intervention. Hospital administrators could combine resilience training and career development support with organizational measures to reduce excessive occupational stressors.

For Hypothesis 3, we found that team job crafting served as a mediator in the relationship between nurses’ stressors and work engagement. Specifically, when nurses experience high stress levels, effective team job crafting was associated with mitigated negative effects of multiple stressors, and this mitigation was associated with improved work engagement. In the JD-R model, individuals skilled in team job crafting may increase their social and structural job resources and may assist in reducing pressures that impede their job capability (Bakker and Demerouti, 2017). For nurses working in a team, achieving quality care involves sharing goal-oriented efforts and crafting jobs collectively, which is associated with increased abilities and resources to handle challenging stressors, and ultimately may promote positive job behavior such as increased work engagement (Iida et al., 2024). In addition, owing to Chinese cultural norms and personal habits, China places a greater emphasis on collectivism and teamwork (Cabrera-Aguilar et al., 2023). Although the team job crafting pathway contributed a smaller share (12% of the total effect) compared to the career resilience pathway, its advantage lies in sustainability and systemic impact – once team-level work patterns are changed, they may yield more lasting positive effects. Therefore, it is meaningful to discuss team job crafting among Chinese nurses. Hospital administrators should focus on the prevalence of team job crafting among nurses.

For Hypothesis 4, this study provided support for the chain-mediating roles of career resilience and team job crafting in the relationship between nurses’ stressors and work engagement. The association indicated that nurses with higher career resilience were more likely to respond to multiple stressors by engaging more in team job crafting; they may utilize cognitive and behavioral changes, proactively mobilize resources, and so on, and this was ultimately associated with increased work engagement. A potential explanation for this result may be that nurses with high career resilience have stronger career-related psychological personal resources to address stressors at work (Chen et al., 2020; Hai-Ping et al., 2020; Harju et al., 2016). Specifically, they maintain a positive attitude toward resolving nurses’ stressors and possess sufficient confidence to counter them; their positive attitude toward their career is also associated with greater self-adjustment in teamwork, enabling the full leverage of available resources to address nurses’ stressors, which was associated with promoting job goals and generating positive outcomes (Bakker, 2010).

Nevertheless, career-related individual differences may not have a uniformly linear effect across all working conditions. For example, previous research has identified curvilinear and context-dependent relationships between career calling and work outcomes, suggesting that the benefits of career-related resources may have thresholds or boundary conditions (Zhou et al., 2020). Moreover, the present mediation analysis did not directly test whether career resilience buffered the association between stressors and work engagement, because such a buffering effect would require testing a stressors * career resilience interaction. Future longitudinal studies could therefore examine potential interaction and quadratic effects and consider organizational conditions such as workload, leadership support, and team climate.

### Implications for practice

Nurses, as the largest occupational group in the healthcare system, are professionals subjected to prolonged exposure to multiple stressors. It is crucial to pay attention to nurses’ work engagement to establish an efficient and sustainable healthcare system. The mediating roles of career resilience and team job crafting in the relationship between nurses’ stressors and work engagement accounted for 46% of the total effect. This indicates that while they are not the sole explanatory mechanisms, they collectively exerted a significant mediating effect. Furthermore, the practical significance of mediation analysis should not be equated with the magnitude of effect sizes. Against the backdrop of a persistent global nursing workforce shortage, even minor, intervention-eligible factors (such as career resilience training and team job crafting interventions) may yield substantial population-level benefits among large cohorts of nurses, which is a fact that cannot be overlooked. These findings of this study suggest that hospital administrators can take the following measures to potentially alleviate nurses’ stress and improve work engagement. First, hospital administrators should pay attention to nurses’ stress levels. Nurses’ stressors should be regularly and dynamically assessed based on individual career cycles to understand their current status. Second, hospital administrators should focus on nurses’ career resilience. The study suggests that nurses can enhance their career resilience by implementing targeted interventions such as career portfolio methods and career challenge consultation platforms, thereby strengthening their positive psychological traits while facing job challenges. Finally, as a teamwork-based profession, nurses should cultivate team job crafting. Hospital administrators can adopt measures – such as teamwork simulation workshops, conducting structured team morning meetings, creating a professional environment that fosters teamwork and supportive communication, and so on – to enhance nurses’ team job crafting and promote positive work behaviors (Aqtam et al., 2024).

### Limitations

This study has several limitations. First, it used a cross-sectional research design that could not infer causal relationships between the variables. Notably, while we tested a sequential mediation model in which career resilience precedes team job crafting, this ordering is a theoretically motivated assumption derived from the JD-R model, rather than an empirically established causal sequence. Future longitudinal studies are required to clarify the potential causal relationships between these variables. Second, the participants were nurses recruited from three tertiary hospitals in Shandong Province using convenience sampling, which may have limited the generalizability of the results. Future studies should recruit participants from other countries or regions to enhance the representativeness of their results. Third, this study used only self-administered questionnaires to collect data, resulting in subjective findings. Future studies should adopt qualitative and quantitative research methods to better explore the factors influencing nurses’ work engagement. Finally, this study only explained the sequential mediating role of career resilience and team job crafting in the relationship between nurses’ stressors and work engagement. Future research should explore the mediating mechanisms of other variables, including organizational factors (such as leadership styles, staffing adequacy, and perceived supervisors’ social support), and work environment factors (such as nurse–patient relationships and shift schedules) to enrich the theoretical model of how nurses’ stressors influence work engagement.

## Conclusion

This study revealed that nurses’ stressors had a direct negative impact on work engagement. Meanwhile, nurses’ stressors influenced work engagement through the mediating effect of career resilience and team job crafting, as well as through the chain-mediating effect involving career resilience and team job crafting. These findings shed light on how nurses’ stressors shape work engagement, helping hospital administrators better understand their teams and design thoughtful, targeted support to foster a more engaged workforce.

## Data Availability

The original contributions presented in the study are included in the article/supplementary material, further inquiries can be directed to the corresponding author.
